# Role of Elm1, Tos3, and Sak1 Protein Kinases in the Maltose Metabolism of Baker’s Yeast

**DOI:** 10.3389/fmicb.2021.665261

**Published:** 2021-06-01

**Authors:** Xu Yang, Lu Meng, Xue Lin, Huan-Yuan Jiang, Xiao-Ping Hu, Cong-Fa Li

**Affiliations:** ^1^College of Food Science and Engineering, Hainan University, Haikou, China; ^2^Engineering Research Center of Utilization of Tropical Polysaccharide Resources, Ministry of Education, Haikou, China; ^3^Hainan Key Laboratory of Food Nutrition and Functional Food, Haikou, China

**Keywords:** Elm1, Tos3, Sak1, maltose metabolism, glucose repression, *Saccharomyces cerevisiae*

## Abstract

Glucose repression is a key regulatory system controlling the metabolism of non-glucose carbon source in yeast. Glucose represses the utilization of maltose, the most abundant fermentable sugar in lean dough and wort, thereby negatively affecting the fermentation efficiency and product quality of pasta products and beer. In this study, the focus was on the role of three kinases, Elm1, Tos3, and Sak1, in the maltose metabolism of baker’s yeast in lean dough. The results suggested that the three kinases played different roles in the regulation of the maltose metabolism of baker’s yeast with differential regulations on *MAL* genes. Elm1 was necessary for the maltose metabolism of baker’s yeast in maltose and maltose-glucose, and the overexpression of *ELM1* could enhance the maltose metabolism and lean dough fermentation ability by upregulating the transcription of *MALx1* (*x* is the locus) in maltose and maltose-glucose and *MALx2* in maltose. The native level of *TOS3* and *SAK1* was essential for yeast cells to adapt glucose repression, but the overexpression of *TOS3* and *SAK1* alone repressed the expression of *MALx1* in maltose-glucose and *MALx2* in maltose. Moreover, the three kinases might regulate the maltose metabolism via the Snf1-parallel pathways with a carbon source-dependent manner. These results, for the first time, suggested that Elm1, rather than Tos3 and Sak1, might be the dominant regulator in the maltose metabolism of baker’s yeast. These findings provided knowledge about the glucose repression of maltose and gave a new perspective for breeding industrial yeasts with rapid maltose metabolism.

## Introduction

Glucose repression is a key regulatory system controlling the synthesis of a series of enzymes involved in the carbohydrate metabolism in yeast (Trumbly, 1992; Klein et al., 1998; Carlson, 1999; Kim et al., 2019). In the presence of glucose, the expression of genes involved in the metabolism of alternate fermentable carbon sources (such as maltose, galactose, and sucrose), non-fermentable ones (such as glycerol, ethanol, and acetate), and respiratory metabolism is repressed (Srđan et al., 2004; Martinez-Ortiz et al., 2019; Lin, 2021). This disadvantage could be substantial in biotechnological production processes, in which glucose is sometimes not the primary carbon source or respiratory metabolism is demanded (Verstrepen et al., 2004). For example, maltose is the most abundant fermentable sugar in lean dough (Hazell and Attfield, 1999; Bell et al., 2001; Jiang et al., 2008). The ability to utilize the maltose in baker’s yeast determines the fermentation efficiency and quality of pasta products (Bell et al., 2001; Jiang et al., 2008). However, glucose represses the expression of genes encoding for maltose permease and maltase, and inhibits the activity of these enzymes in baker’s yeast, thereby, negatively affecting the maltose metabolism and lean dough fermentation of baker’s yeast (Srđan et al., 2004; Hatanaka et al., 2009; Sun et al., 2012; Zhang et al., 2015b,c). Thus, alleviating glucose repression is essential to improve the utilization efficiency of non-glucose carbon sources.

The Snf1 protein kinase is a member of the remarkably conserved AMP-activated protein kinase (AMPK) family in eukaryotes (Meng et al., 2021). The Snf1 kinase is crucial to the glucose derepression of *Saccharomyces cerevisiae*, ensuring the availability of non-preferred carbon sources (Coccetti et al., 2018; Persson et al., 2020). Snf1 regulates the expression of glucose-repressed genes by regulating the phosphorylation status and nuclear localization of the repressor Mig1 (Östling and Ronne, 1998). In glucose limitation, Snf1 is activated, and phosphorylates repressor Mig1, thereby preventing the interaction of Mig1 with the corepressors Ssn6-Tup1 and promoting the transcription of downstream glucose-repressed genes (Östling and Ronne, 1998; Papamichos-Chronakis et al., 2004). Unphosphorylated Mig1 is retained in the nucleus, and interacts with Ssn6-Tup1 when Snf1 is inactive in high glucose (Östling and Ronne, 1998; Papamichos-Chronakis et al., 2004).

The Snf1 protein kinase is a complex that consists of an alpha catalytic subunit Snf1, a gamma regulatory subunit Snf4, and one of the three alternative beta regulatory subunits, namely, Sip1, Sip2, and Gal83 (Daniel and Carling, 2002; García-Salcedo et al., 2014; Meng et al., 2020). Snf1 activation requires at least two steps: First, Snf4 binds to the C-terminal regulatory domain of the catalytic subunit Snf1 to counteract the autoinhibition of Snf1, in which β regulatory subunits participate in the linkage of Snf1 and Snf4, and direct the subcellular localization of Snf1 (McCartney and Schmidt, 2001). Second, the phosphorylation of the Thr210 site of the catalytic subunit Snf1 is initiated by three upstream protein kinases, namely, Elm1, Tos3, and Sak1 (Hong et al., 2003; García-Salcedo et al., 2014). The three kinases exhibit a stress-dependent demand for the activation of different isoforms of Snf1, and contribute differently to cellular regulation in various carbon sources (McCartney et al., 2005). Although Sak1 appears to be the major one in the Snf1-dependent regulation of the metabolism of non-preferred carbon sources such as raffinose, ethanol, and glycerol, any of the three kinases is sufficient to activate Snf1 (Hedbacker et al., 2004; Liu et al., 2011). The *sak1*Δ mutants of *Candida albicans* fail to grow on many alternative carbon sources (Ramírez-Zavala et al., 2017). Tos3 is more important in the activation of Snf1 in non-fermentable carbon sources than in an abrupt glucose stress (Kim et al., 2005). The single mutation of *ELM1* does not display an Snf1 phenotype in raffinose, but *SAK1*, *TOS3*, and *ELM1* triple deletions do (Sutherland et al., 2003). However, the role of the three upstream kinases in maltose metabolism is unclear.

In the current study, the genes *ELM1*, *TOS3*, and *SAK1* were overexpressed and deleted in baker’s yeast ABY3α alone to explore the role of the kinases Elm1, Tos3, and Sak1 in the maltose metabolism of baker’s yeast. The growth characteristic, maltose utilization, lean dough leavening ability, and mRNA level of genes related to the maltose metabolism of the strains were analyzed.

## Materials and Methods

### Strains and Plasmids

The strains and plasmids used in the current work were listed in Table 1.

**TABLE 1:** Characteristics of strains and plasmids used in the current study.

**Strains or plasmids**	**Relevant characteristic**	**Reference or source**
**Strains**		
*Escherichia coli* DH5α	Φ80 *lacZΔM15 ΔlacU169 recA1 endA1 hsdR17 supE44 thi-1 gyrA relA1*	This lab
*S. cerevisiae* ABY3α	Industrial baker’s yeast haploid strain	This lab
A+YP	Yep-PK	This study
A+E	Yep-PEK	This study
A+T	Yep-PTK	This study
A+S	Yep-PSK	This study
A-E	*MAT α*, Δ*elm1*:: *loxP*	This study
A-T	*MAT α*, Δ*tos3*:: *loxP*	This study
A-S	*MAT α*, Δ*sak1*:: *loxP*	This study
A-SNF1	*MAT α*, Δ*snf1*:: *loxP*	This study
A+PK-SNF1	*MAT α*, Yep-PK, Δ*snf1*:: *loxP*	This study
A-REG1	*MAT α*, Δ*reg1*:: *loxP*	This study
A+PK-REG1	*MAT α*, Yep-PK, Δ*reg1*:: *loxP*	This study
A+E-SNF1	*MAT α*, Yep-PEK, Δ*snf1*:: *loxP*	This study
A+E-REG1	*MAT α*, Yep-PEK, Δ*reg1*:: *loxP*	This study
A+T-REG1	*MAT α*, Yep-PTK, Δ*reg1*:: *loxP*	This study
A+S-REG1	*MAT α*, Yep-PSK, Δ*reg1*:: *loxP*	This study
**Plasmids**		
pUG6	*E. coli/S. cerevisiae shuttle vector*, *containing Amp^+^*, *loxP-KanMX-loxP* disruption cassette	This lab
pSH-Zeocin	Zeo*^*r*^*, Cre expression vector	This lab
Yep-P	*URA3*^+^, *Amp^*R*^ ori* control vector, *PGK1_*P*_-PGK1_*T*_*	Gifted by Zhang et al. (2015a)
Yep-PK	*KanMX*, *PGK1_*P*_-PGK1_*T*_*	This study
Yep-PEK	*KanMX*, *PGK1_*P*_-ELM1-PGK1_*T*_*	This study
Yep-PTK	*KanMX*, *PGK1_*P*_-TOS3-PGK1_*T*_*	This study
Yep-PSK	*KanMX*, *PGK1_*P*_-SAK1-PGK1_*T*_*	This study

### Media and Culture Conditions

The *E. coli* strains were cultured in the Luria-Bertani medium (10 g/L of tryptone, 10 g/L of NaCl, and 5 g/L of yeast extract) at 37°C, and 100 mg/L of ampicillin was used for selecting the positive *E. coli* transformants. The yeast strains were cultured in the yeast extract peptone dextrose (YEPD) medium that contains 20 g/L of glucose, 20 g/L of peptone, and 10 g/L of yeast extract. Maltose fermentation was conducted in the low sugar model liquid dough (LSMLD) medium, in which 33.25 g/L of maltose mixed with 5 g/L of glucose or 38 g/L of maltose was used as the carbon source, according to the previous study (Lin et al., 2018).

The yeast strains, which were preserved on slopes at the exponential phase, were inoculated into the YEPD medium by an inoculating loop to the stationary stage at 30°C. Then, 10% of the cultures were inoculated to the YEPD medium at 30°C with 180 rpm rotary shaking for 24 h. The second-cultures were centrifugated at 4°C with 1,500 × *g* for 5 min. The cells were collected after washing with distilled water twice.

### Construction of Plasmids and Transformants

Yeast genomic DNA was obtained using the yeast DNA kit (D1900, Solarbio, Beijing, China). Plasmids were obtained using the Plasmid Mini Kit II (DC201-01, Vazyme, Jiangsu, China). The gene fragments were cloned to the plasmids using the ClonExpressIIOne Step Cloning Kit (C112, Vazyme, Jiangsu, China). Primers used in this work were listed in Table 2.

**TABLE 2:** Primers used in the present study.

**Primer**	**Sequence (5′ → 3′)**
**For genes overexpression**	
ELM1-F	CAAGATCGGAATTCCAGATCTATGTCACCTCGACA GCTTATACCG
ELM1-R	ATCTATCGCAGATCCCTCGAGCTATATTTGACCATTA TCTGCAAAGTTC
TOS3-F	CAAGATCGGAATTCCAGATCTATGGTACTACTTAAAG AACCTGTTCAGC
TOS3-R	ATCTATCGCAGATCCCTCGAGCTAAAGCTTATAAAG AGACATTCTTCTTCTC
SAK1-F	CAAGATCGGAATTCCAGATCTATGGATAGGAGTGAT AAAAAAGTTAACG
SAK1-R	ATCTATCGCAGATCCCTCGAGTCATGGAAGTGCACT CCTTCTCT
Kan-F	AGAGTCGACCTGCATGCCAGCTGAAGCTTCGTACG CTG
Kan-R	GCCAGTGCCAAGCTTGCATGCGCATAGGCCACTAG TGGATCTGA
**For genes deletion**	
ELM1-BA-F	ACGCTGCCTTATCCATTGACCGAG
ELM1-BA-R	TCCTGCAGCGTACGAAGCTTCAGCTGTTCATGCTAA GTAATTATTGTTAAC
ELM1-Kan-F	GTTAACAATAATTACTTAGCATGAACAGCTGAAGCTT CGTACGCTGCAGG
ELM1-Kan-R	GACAGATATCATCCTGTAGTTTCATGCATAGGCCAC TAGTGGATCTGATA
ELM1-BB-F	TATCAGATCCACTAGTGGCCTATGCATGAAACTACA GGATGATATCTGTC
ELM1-BB-R	ATGAGTTCGCACTGGTGCAGGTAC
TOS3-BA-F	AGGTCAAGACGAAAACCATAAATA
TOS3-BA-R	CCTGCAGCGTACGAAGCTTCAGCTGATTCTTCAAA GCTTCCTTTTTATAT
TOS3-Kan-F	ATATAAAAAGGAAGCTTTGAAGAATCAGCTGAAGCT TCGTACGCTGCAGG
TOS3-Kan-R	ATTAAAATAATTTACATATATCATGGCATAGGCCACT AGTGGATCTGATA
TOS3-BB-F	TATCAGATCCACTAGTGGCCTATGCCATGATATATGT AAATTATTTTAAT
TOS3-BB-R	GATTTTACGAATGCCTATGGTGAC
SAK1-BA-F	CGAACGATACCTCAAGGAGCAAGA
SAK1-BA-R	CCTGCAGCGTACGAAGCTTCAGCTGGTTCAAAACT CCTTATTAATATGCT
SAK1-Kan-F	AGCATATTAATAAGGAGTTTTGAACCAGCTGAAGCTT CGTACGCTGCAGG
SAK1-Kan-R	ATGGAAATTACTTTGAATTTTACACGCATAGGCCACT AGTGGATCTGATA
SAK1-BB-F	TATCAGATCCACTAGTGGCCTATGCGTGTAAAATTCA AAGTAATTTCCA
SAK1-BB-R	AAGCTGGTGGGAAATAACAAGGAT
SNF1-K-F	GAAGTTTTTTTTTGTAACAAGTTTTGCTACACTCCCT TAATAAAGTCAACCAGCTGAAGCTTCGTACGC
SNF1-K-R	CCCAGCCGTCAAATTTGAAATCCACCAAATAATTATT GGTTGCATAGGCCACTAGTGGATCTG
REG1-K-F	TGACGAAGACGAGATAAGAAAAATCCAAAACAGCT GAAGCTTCGTACGC
REG1-K-R	TTCATGTTGACTTCAAAATTCTTTCTTGCATAGGCCA CTAGTGGATCTG
**For verification**	
PGK-F	TCTAACTGATCTATCCAAAACTGA
PGK-R	TAACGAACGCAGAATTTTC
Kan-FV	CAGCTGAAGCTTCGTACGC
Kan-RV	GCATAGGCCACTAGTGGATCTG
ELM1-U1	CTGGTCGTAGCCACATAACCGTTCC
ELM1-D1	TCGTCATCAAAATCACTCGCATCA
ELM1-U2	GCGTTGCCAATGATGTTACAGATG
ELM1-D2	ATCCTACCAGATACGCTTCGCTTG
TOS3-U1	TTTAGTTAGTTTCTTCATCGTTCG
TOS3-D1	CAGCCAGTTTAGTCTGACCATCTCAT
TOS3-U2	ATGCTGGTCGCTATACTGCTGTCG
TOS3-D2	AGAAGAACAAGACTCAGACGATGC
SAK1-U1	ACTGATACATCTCCACAGGCTAAG
SAK1-D1	GATAAAATGCTTGATGGTCGGAAG
SAK1-U2	GCTGGTCGCTATACTGCTGTCGAT
SAK1-D2	CTCTTTTACCACTGTGCCCCAATC
S-F	GGCTGTTTCAATAATCATAGCGAAAGAAATA
S-R	CCGTCAAATTTGAAATCCACCAAATAATTATTG
R-F	GGCTGTTATACGTATAACCACACAC
R-R	CTTCGCTGTCTACATTTGTCCTTGA
K-F	CTTGCTAGGATACAGTTCTCACATCA
K-R	CGCATCAACCAAACCGTTATTCATTC
Z-F	CCCACACACCATAGCTTCA
Z-R	AGCTTGCAAATTAAAGCCTT
**For RT-PCR**	
ACT1-F	ATTGATAACGGTTCTGGT
ACT1-R	AATTGGGTAACGTAAAGTC
MAL61-F	TACCTCCGTTTGTTTGCG
MAL61-R	AGGACCATTGTGAGACCC
MAL62-F	AGTTTCCTGGCAAATCGG
MAL62-R	GTCCCACGGCAATCATAC
MAL31-F	TCCCAGAACAAATATGCCAACT
MAL31-R	TCTCGGGTCCTTTACCACTTAA
MAL32-F	TCCAGAAACAGAACCAAAGTGG
MAL32-R	AGTCATAAAACGGACAAACCCA

To construct the episomal plasmid Yep-PEK, firstly, the gene *ELM1* was amplified using the primers ELM1-F/ELM1-R with the genome of ABY3α as a template. Secondly, the fragment of *ELM1* was inserted into the *Xho*I site of the promoter and terminator of *PGK1* in the plasmid Yep-P, yielding the plasmid Yep-PE. Finally, the selectable marker fragment *KanMX*, which was amplified using the primers Kan-F/Kan-R with the vector pUG6 as a template, was inserted into the *Sph*I site of the plasmid Yep-PE. The episomal plasmids Yep-PTK and Yep-PSK were constructed using the abovementioned strategy with the primers TOS3-F/TOS3-R and SAK1-F/SAK1-R, respectively.

To obtain the *ELM1*-deleted mutant, the method ‘DNA assembler’ was used to rapidly assemble the fragments on the chromosome (Shao et al., 2008; Li et al., 2018). The upstream and downstream sequences of *ELM1* were amplified using the primers ELM1-BA-F/ELM1-BA-R, and ELM1-BB-F/ELM1-BB-R, respectively, with the genome of ABY3α as a template. The selectable marker gene *KanMX* was amplified using the primers ELM1-Kan-F/ELM1-Kan-R with the vector pUG6 as a template. The three fragments were transformed into the strain ABY3α, and *KanMX* was integrated to the *ELM1* site of ABY3α by homologous recombination. The *TOS3*-deleted and *SAK1*-deleted mutants were constructed using the same strategy with the primers TOS3-BA-F/TOS3-BA-R, TOS3-BB-F/TOS3-BB-R, and TOS3-Kan-F/TOS3-Kan-R and SAK1-BA-F/SAK1-BA-R, SAK1-BB-F/SAK1-BB-R, and SAK1-Kan-F/SAK1-Kan-R, respectively.

To obtain the *SNF1*-deleted mutant, firstly, the selectable marker gene *KanMX* was amplified using the primers SNF1-Kan-F/SNF1-Kan-R with the vector pUG6 as a template. Secondly, the fragment SNF1F-Kan-SNF1R was integrated to the *SNF1* fragment site of ABY3α. Finally, the gene *KanMX* was knocked out by the Cre/*Lox* system. The *REG1*-deleted mutant was constructed using the same strategy with the primers REG1-Kan-F/REG1-Kan-R.

Yeast transformations were obtained using the lithium acetate/PEG method (Gietz, 2015). A total of 800 mg/L of G418 (Promega, Madison, WI, United States) was used to select the positive *S. cerevisiae* transformants. The YEPG medium (20 g/L of galactose, 20 g/L of peptone, and 10 g/L of yeast extract) was used for the Cre expression in the yeast transformants. A total of 500 mg/L of Zeocin (R25001, Invitrogen, Carlsbad, CA, United States) was used to select the yeast strains carrying the plasmid pSH-Zeocin. The plasmids Yep-PK, Yep-PEK, Yep-PTK, and Yep-PSK were transformed into the parental strain to obtain the transformants A+YP, A+E, A+T, and A+S, respectively. The plasmid Yep-PEK was transformed into the *SNF1*-deleted mutant to get the transformant A+E-SNF1. The plasmids Yep-PEK, Yep-PTK, and Yep-PSK were transformed into the *REG1*-deleted mutant to get the transformants A+E-REG1, A+T-REG1, and A+S-REG1, respectively. The primers used in the verification of the strains were listed in Table 2. The fragment containing *PGK1* and the overexpressed gene was verified by PCR to confirm the transformation of an episomal plasmid. The fixed-point verification method, which used the primers that were intercepted from the outside of the upstream/downstream homologous of the target gene and from the inside of the *KanMX* gene, was used to verify the gene-deleted mutants. The amplification of *Zeocin* was used to verify the transformation of the plasmid pSH-Zeocin.

### Measurement of Growth Curve

Yeast cells were inoculated in the 2% glucose and 2% maltose conditions at 30°C. Then, the 3% (vol/vol) inoculations (equivalent to 3 × 10^8^ cells) were transferred to the same condition, and the cell density OD_600_ was monitored at 30°C using a UV spectrophotometer (T6, Persee, Beijing, China). The specific growth rate was calculated by the change in the OD_600_ Napierian logarithm versus time during exponential growth. A total of 10 mL of the cell culture was filtered in the stationary phase, washed twice with 10 mL of distilled water, and then dried at 105°C for 24 h to measure the cell dry weight. The biomass yield was expressed as the gram (dry weight) of yeast cells per liter of medium. Experiments were conducted thrice.

### Measurement of Extracellular Sugar

A total of 2 g of fresh yeast was cultured in the LSMLD media at 30°C. The cultures were sampled at different fermentation time points. The measurement of extracellular sugars and the calculation of maltose utilization efficiency and time span value were conducted using the previous method (Lin et al., 2018). The samples were filtered through 0.45 μm pore size cellulose acetate filters (Millipore Corp, Danvers, MA, United States) and analyzed by high-performance liquid chromatography with a refractive index detector and a SilGreen R GH0830078H column (300^∗^7.8 mm^∗^8 um, SilGreen, Beijing, China) at 65°C. 5 mM H_2_SO_4_ was used as the mobile phase at a flow rate of 0.6 mL/min.

The maltose utilization efficiency in the maltose LSMLD medium was determined by the ratio of the consumed maltose in the whole process to the total maltose. The maltose utilization efficiency in the maltose-glucose LSMLD medium was determined by the ratio of the consumed maltose when glucose was exhausted to the total maltose. The time span refers to the difference between the time points at which half of the maltose and half of the glucose was consumed in the maltose-glucose LSMLD medium. Three independent experiments were performed.

### Test of CO_2_ Production

The leavening ability of yeast cells in lean dough was tested according to the previous study (Zhang et al., 2015b), based on the Chinese National Standards for yeast used in food processing with the following modification. First, 140 g of standard flour, 72.5 mL of water, 4.5 g of fresh yeast, and 2 g of salt were evenly and quickly mixed at 30 ± 0.2°C of the dough center temperature in 5 min. Then, 50 g of mixed lean dough was transferred to a 250 mL graduated cylinder and fermented at 30°C. CO_2_ amounts were measured by the change of dough height in 120 min. Three independent experiments were carried out.

### Real-Time Quantitative PCR (RT-qPCR)

Two grams (2 g) of fresh yeast was cultured in the LSMLD media, and 1 mL cultures (equivalent to 5 × 10^6^ cells) were sampled at 30 min. The expression levels of the genes *MAL61/MAL31* encoding maltose permease and *MAL62/MAL32* encoding maltase were tested according to the previous study (Lin et al., 2018). The total cellular RNA was extracted using an RNA-eazy isolation reagent (R701, Vazyme, Jiangsu, China). Using mRNA as a template, cDNA was synthesized using a HIScript III RT SuperMix for qPCR (+gDNA wiper) (R323-01, Vazyme, Jiangsu, China). Changes in the expression levels of *MAL* genes were assessed through qRT-PCR with a ChamQ Universal SYBR qPCR Master Mix (Q711-02, Vazyme, Jiangsu, China). Actin was used as the loading control. The primers used for amplifying the target genes and the reference gene *ACT1* were shown in Table 2. The expression level of the target genes in the parental strain ABY3α was normalized to the reference gene. Experiments were conducted thrice.

### Statistical Analysis

Student’s *t*-test was performed to analyze the differences of the transformants and the parental strain. Differences at *P <* 0.05 were considered as statistically significant differences.

## Results

### Growth Property

**FIGURE 1:**
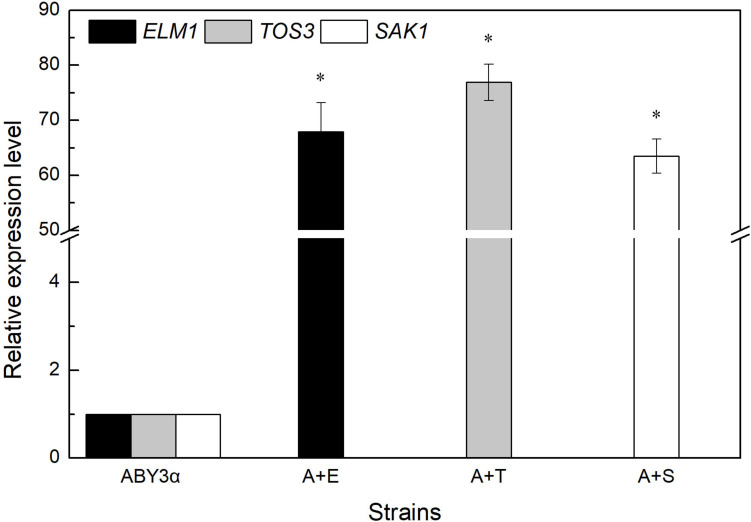
mRNA level of the overexpressed genes. The expression level of *ELM1* in the strain A+E, *TOS3* in the strain A+T, and *SAK1* in the strain A+S was tested using qRT-PCR. The cells were sampled from YEPD medium at 16 h. ABY3α: the parental strain; A+E: the transformant carrying *ELM1* overexpression; A+T: the transformant carrying *TOS3* overexpression; A+S: the transformant carrying *SAK1* overexpression. Significant differences of the transformants to the parental strain were confirmed at *p < 0.05.

**FIGURE 2:**
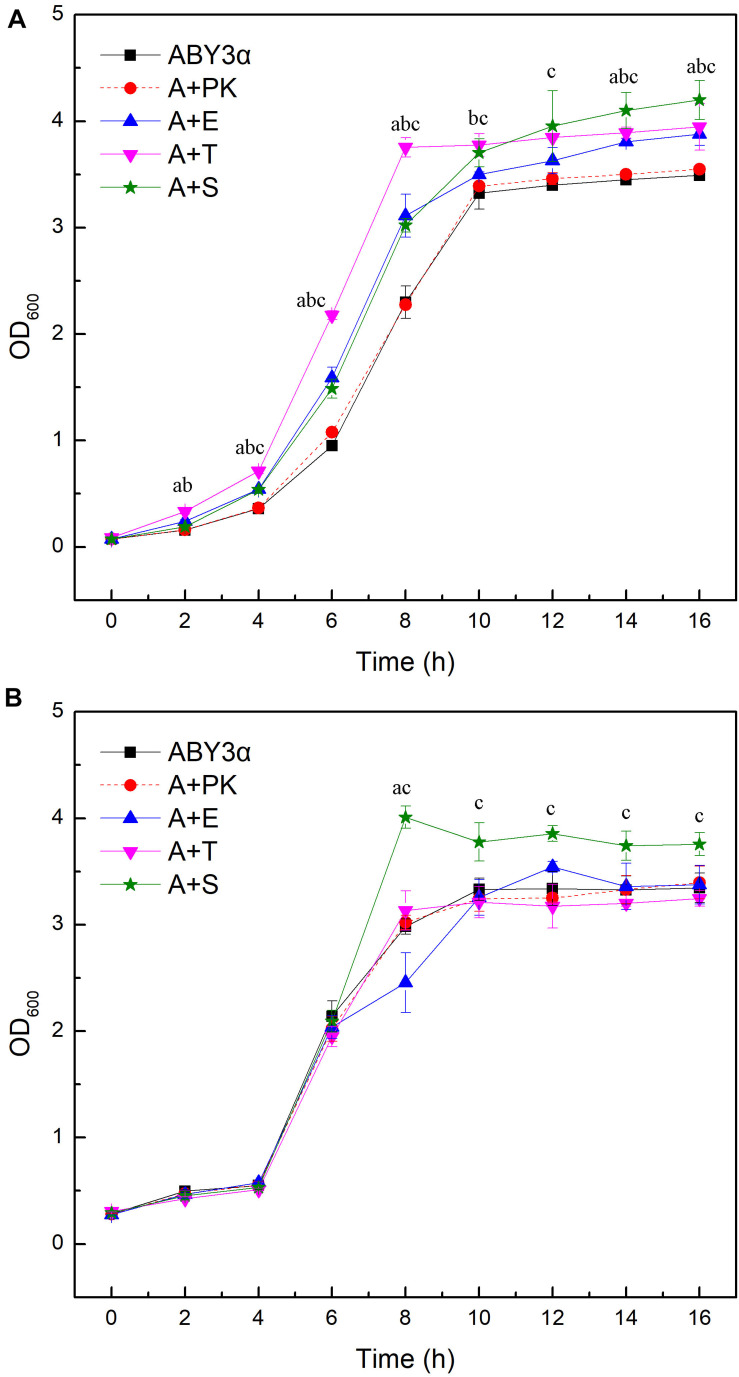
Growth curves of the strains carrying genes overexpression. Growth curves were monitored in **(A)** 2% glucose (YEPD medium) and **(B)** 2% maltose (YEPM medium consisted of 20 g/L maltose, 20 g/L peptone, and 10 g/L yeast extract) conditions at appropriate time intervals at 30°C. ABY3α, the parental strain; A+PK, the strain carrying the vector Yep-PK used as a blank control to demonstrate any possible effect of the empty vector; A+E, the transformant carrying *ELM1* overexpression; A+T, the transformant carrying *TOS3* overexpression; A+S, the transformant carrying *SAK1* overexpression. Significant differences of the transformants A+E, A+T, and A+S to the parental strain were confirmed at ^a^*p* < 0.05, ^b^*p* < 0.05, and ^c^*p* < 0.05, respectively.

Under the control of the constitutive yeast phosphoglycerate kinase gene (*PGK1*) promoter (*PGK1*_*P*_) and terminator (*PGK1*_*T*_), the mRNA expression level of *ELM1*, *TOS3*, and *SAK1* were upregulated by 68-, 77-, and 64-fold in the transformants A+E, A+T, and A+S, respectively, compared with the parental strain (Figure 1). To test the influence of *ELM1*/*TOS3*/*SAK1* overexpression on the growth characteristic of baker’s yeast, the growth curves of the strains were monitored in 2% glucose and maltose (Figure 2). Moreover, the specific growth rate and biomass yield of the strains were calculated (Table 3). Transformant A+YP, a blank control to reflect any possible influence of an empty vector, displayed growth similar to that of the parental strain. *ELM1*-overexpressed transformant A+E, *TOS3*-overexpressed transformant A+T, and *SAK1*-overexpressed transformant A+S improved cell proliferation to varying degrees in glucose. In maltose, compared with parental strain ABY3α, the specific growth rate of the transformant A+E decreased from 0.460 to 0.423 h^–1^ in maltose, but the final biomass yield showed a slight change. Only the transformant A+S exhibited an enhanced specific growth rate and biomass yield in maltose. Compared with the parental strain, although obvious changes of the specific growth rate were observed in the gene-deleted mutants (Supplementary Table 1, Supplementary Figure 1), the deletion of any of the three genes did not influence the final biomass yield in glucose; the deletions of *ELM1* and *SAK1* inhibited the biomass yield in maltose instead.

**TABLE 3:** Specific growth rate (h^–1^) and biomass yield (g/L) of the strains carrying genes overexpression.

	**Glucose**		**Maltose**	
**Strains**	**Specific growth rate**	**Biomass yield**	**Specific growth rate**	**Biomass yield**
ABY3α	0.512 ± 0.002	8.24 ± 0.12	0.460 ± 0.002	8.08 ± 0.09
A+PK	0.514 ± 0.003	8.27 ± 0.11	0.454 ± 0.003	8.11 ± 0.08
A+E	0.520 ± 0.002	9.04 ± 0.08*	0.423 ± 0.005*	8.30 ± 0.11
A+T	0.539 ± 0.003*	8.88 ± 0.09*	0.465 ± 0.003	8.14 ± 0.09
A+S	0.551 ± 0.004*	9.36 ± 0.10*	0.583 ± 0.003*	8.94 ± 0.09*

*ABY3α, the parental strain; A+PK, the strain carrying the vector Yep-PK used as a blank control to demonstrate any possible effect of the empty vector; A+E, the transformant carrying *ELM1* overexpression; A+T, the transformant carrying *TOS3* overexpression; A+S, the transformant carrying *SAK1* overexpression.*

Significant differences of the transformants to the parental strain were confirmed at *p < 0.05.

These results demonstrated that the three kinases had a redundancy function in the cell growth of baker’s yeast in glucose. However, increasing each gene dosage was sufficient to enhance cell growth in glucose, and only an increased *SAK1* level could promote cell growth in maltose. Therefore, the three kinases, Elm1, Tos3, and Sak1, performed a different regulation of the baker’s yeast growth via a carbon source-dependent pathway.

### Sugar Consumption in the LSMLD Media

**FIGURE 3:**
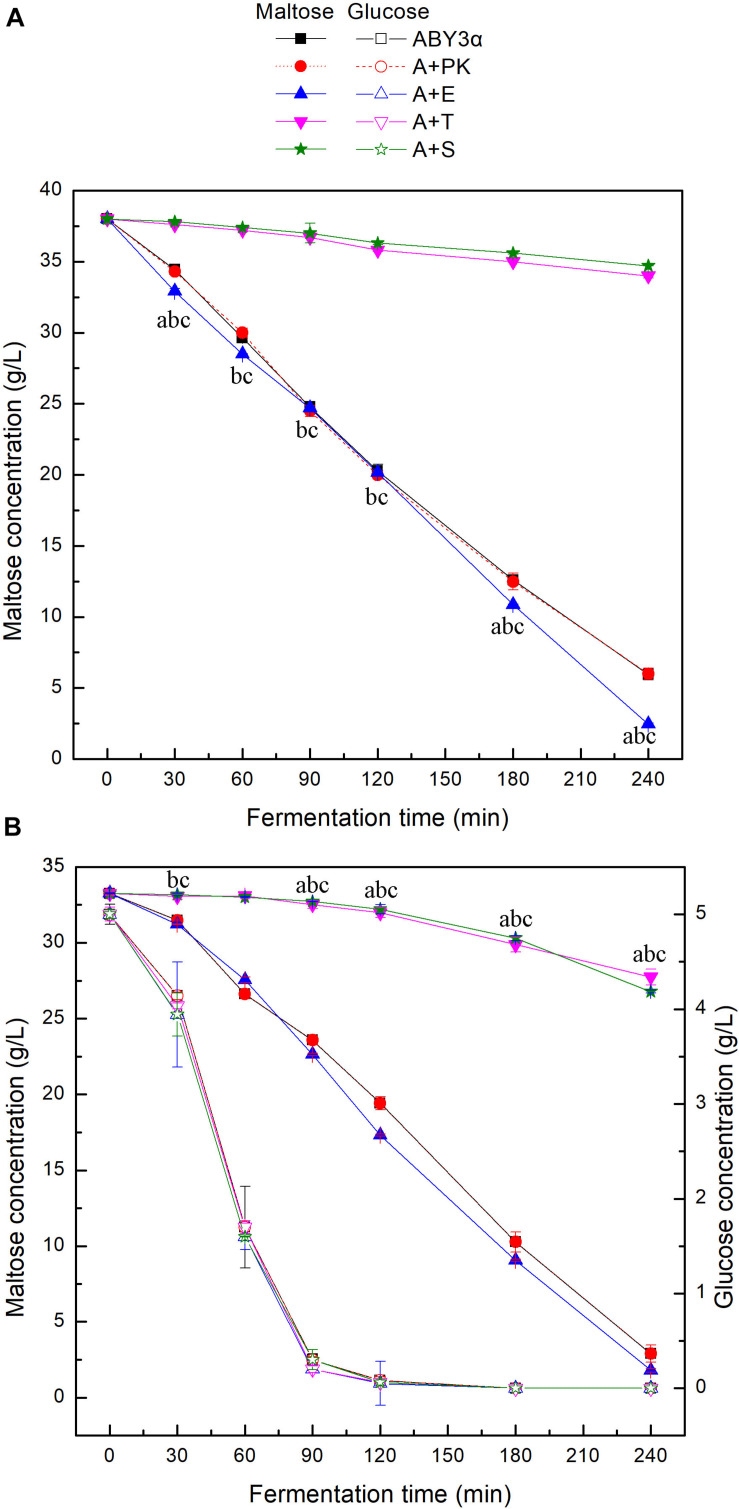
Sugar consumption of the strains carrying genes overexpression in LSMLD media. 2 g fresh yeast was cultured in the **(A)** maltose and **(B)** maltose-glucose LSMLD media at 30°C and 1 mL cultures were sampled at a certain interval. ABY3α, the parental strain; A+PK, the strain carrying the vector Yep-PK used as a blank control to demonstrate any possible effect of the empty vector; A+E, the transformant carrying *ELM1* overexpression; A+T, the transformant carrying *TOS3* overexpression; A+S, the transformant carrying *SAK1* overexpression. Significant differences of the transformants A+E, A+T, and A+S to the parental strain were confirmed at ^a^*p* < 0.05, ^b^*p* < 0.05, and ^c^*p* < 0.05, respectively.

To investigate the influence of *ELM1*/*TOS3*/*SAK1* overexpression on the maltose metabolism of baker’s yeast, the utilization of maltose and glucose was tested in the LSMLD media (Figure 3). Blank control strain A+YP exhibited sugar consumption similar to that of the parental strain ABY3α. Compared with the parental strain ABY3α, *ELM1* overexpression strain A+E increased the maltose utilization efficiency by 15 and 11% in the maltose-glucose and maltose LSMLD media, respectively, and no evident changes of glucose utilization were observed in the maltose-glucose condition. Simultaneously, the deletion of *ELM1* repressed the maltose consumption in maltose-glucose and maltose (Supplementary Figure 2). Time span value, a parameter that judges the degree of glucose repression, was calculated from Figure 3B. An 18% decrease (1.65 h in the parental strain and 1.35 h in the strain A+E) of the time span was obtained in the transformant A+E compared with the parental strain. Unexpectedly, *TOS3* overexpression strain A+T and *SAK1* overexpression strain A+S showed a much lower maltose utilization than the parental strain in the maltose-glucose and maltose conditions, with no noticeable difference in glucose consumption. The single deletion of *TOS3* and *SAK1* did not affect maltose consumption in maltose, whereas, a negative effect was observed in maltose-glucose (Supplementary Figure 2).

These findings reflected that Elm1 might be the positive regulator of the maltose metabolism in baker’s yeast used in this work in the analyzed conditions. Tos3 and Sak1 were not necessary for maltose metabolism in maltose, and even negatively regulated the maltose metabolism at a high expression level. Nevertheless, native expression levels of *TOS3* and *SAK1* were essential for the baker’s yeast cell to resist glucose repression to utilize maltose in the maltose-glucose condition.

### Fermentation Property in Lean Dough

**FIGURE 4:**
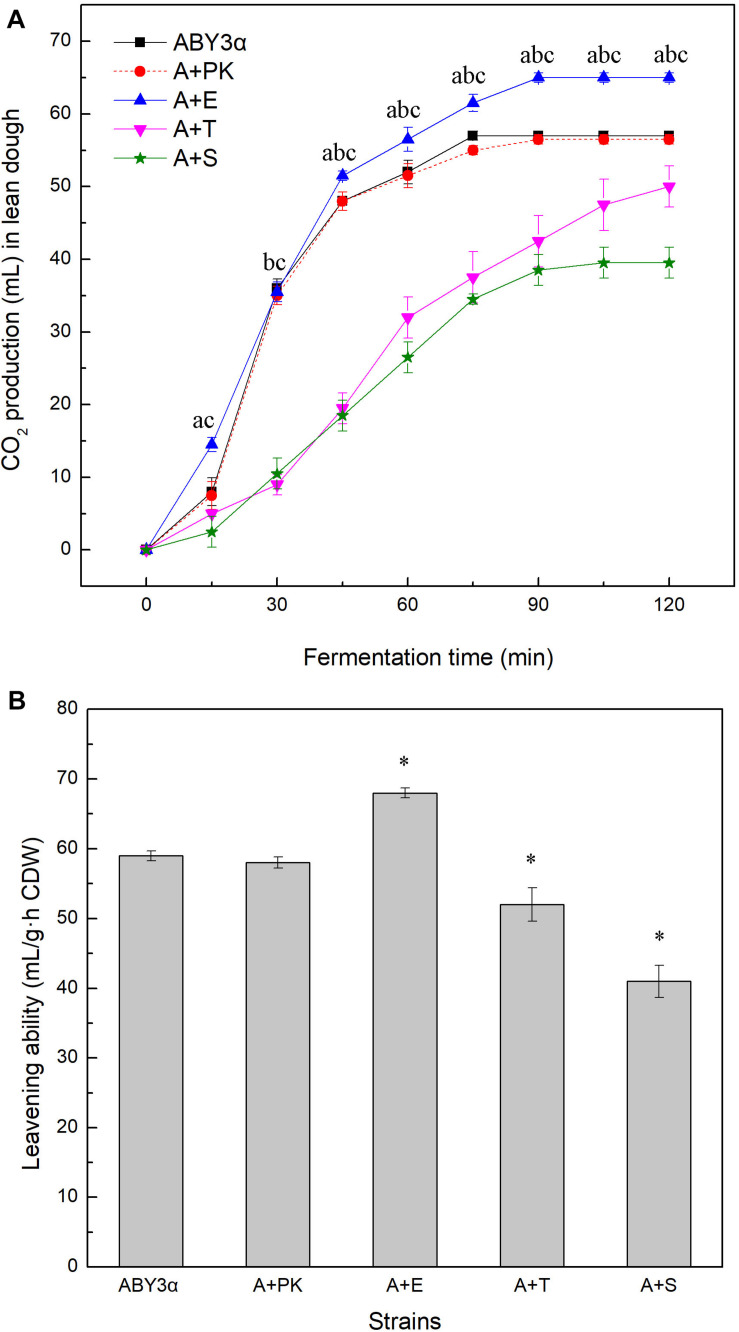
CO_2_ production of the strains carrying genes overexpression in lean dough. **(A)** Mixed dough was placed into a graduated cylinder, and CO_2_ amounts were recorded at 30°C for 120 min. Significant differences of the transformants A+E, A+T, and A+S to the parental strain were confirmed at ^a^*p* < 0.05, ^b^*p* < 0.05, and ^c^*p* < 0.05, respectively. **(B)** Leavening ability was determined by CO_2_ production per hour per gram (dry weight) of yeast cells. Significant differences of the transformants to the parental strain were confirmed at ^∗^*P* < 0.05. ABY3α, the parental strain; A+PK, the strain carrying the vector Yep-PK used as a blank control to demonstrate any possible effect of the empty vector; A+E, the transformant carrying *ELM1* overexpression; A+T, the transformant carrying *TOS3* overexpression; A+S, the transformant carrying *SAK1* overexpression.

The fermentation capacity of the transformants and the parental strain was measured in lean dough to further test the influence of *ELM1*/*TOS3*/*SAK1* overexpression on the maltose metabolism of baker’s yeast (Figure 4). Blank control strain A+YP exhibited a fermentation performance similar to that of the parental strain ABY3α. *ELM1* overexpression strain A+E exhibited a stronger CO_2_-releasing ability than the parental strain. Compared with the parental strain ABY3α, the transformant A+E increased the total amounts of CO_2_ within 120 min and leavening ability by 12 and 15%, respectively; decreases of 21 and 26% were observed in the *ELM1* deletion (Supplementary Figure 3). On the contrary, the total amounts of CO_2_ within 120 min and the leavening power of *TOS3* overexpression strain A+T were 12 and 23% lower than those of the parental strain, respectively; those for *SAK1* overexpression strain A+S were 30 and 21% lower, respectively.

The single deletion of *TOS3* and *SAK1* delayed the release of CO_2_ in the early stage of fermentation, but this inhibition effect might be relieved with the release of glucose repression in lean dough.

These results confirmed the positive effect of *ELM1* overexpression on maltose metabolism of baker’s yeast and suggested the importance of the normal transcription of *TOS3* and *SAK1* in the lean dough fermentation.

### Expression Level of *MAL* Genes

**FIGURE 5:**
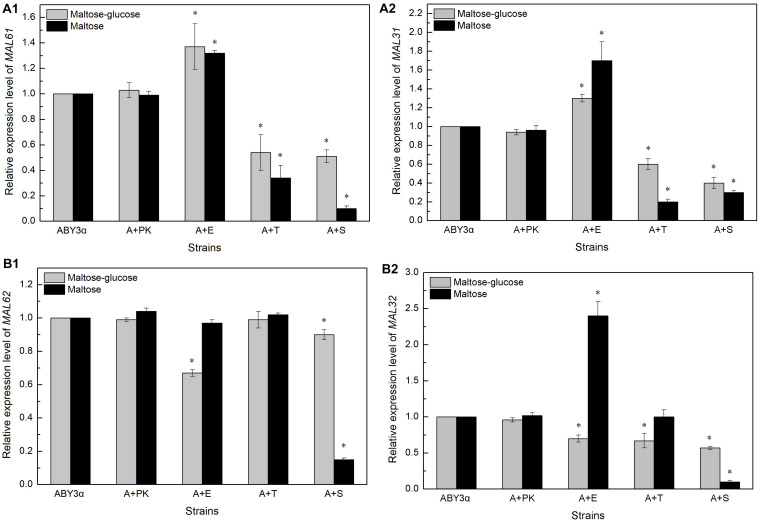
Expression levels of **(A)** *MALx1* and **(B)** *MALx2* in the single-kinase gene overexpression. 2 g fresh yeast was cultured in the LSMLD media and cultures were sampled at 30 min. The expression levels of genes **(A1)** *MAL61*, **(A2)** *MAL31* and **(B1)** *MAL62*, **(B2)** *MAL32* were tested using qRT-PCR. ABY3α, the parental strain; A+PK, the strain carrying the vector Yep-PK used as a blank control to demonstrate any possible effect of the empty vector; A+E, the transformant carrying *ELM1* overexpression; A+T, the transformant carrying *TOS3* overexpression; A+S, the transformant carrying *SAK1* overexpression. Significant differences of the transformants to the parental strain were confirmed at ^∗^ *P* < 0.05.

The transcription of genes *MAL61/MAL31* and *MAL62/MAL32* was analyzed in the maltose-glucose and maltose conditions (Figure 5, Supplementary Figure 4). In general, Elm1 positively regulated the transcription of *MAL61* and *MAL31* in maltose and maltose-glucose and the transcription of *MAL62* and *MAL32* in maltose. By contrast, Tos3 and Sak1 negatively regulated the expression of *MAL61*/*MAL31* in maltose-glucose and the expression of *MAL32* in maltose.

**FIGURE 6:**
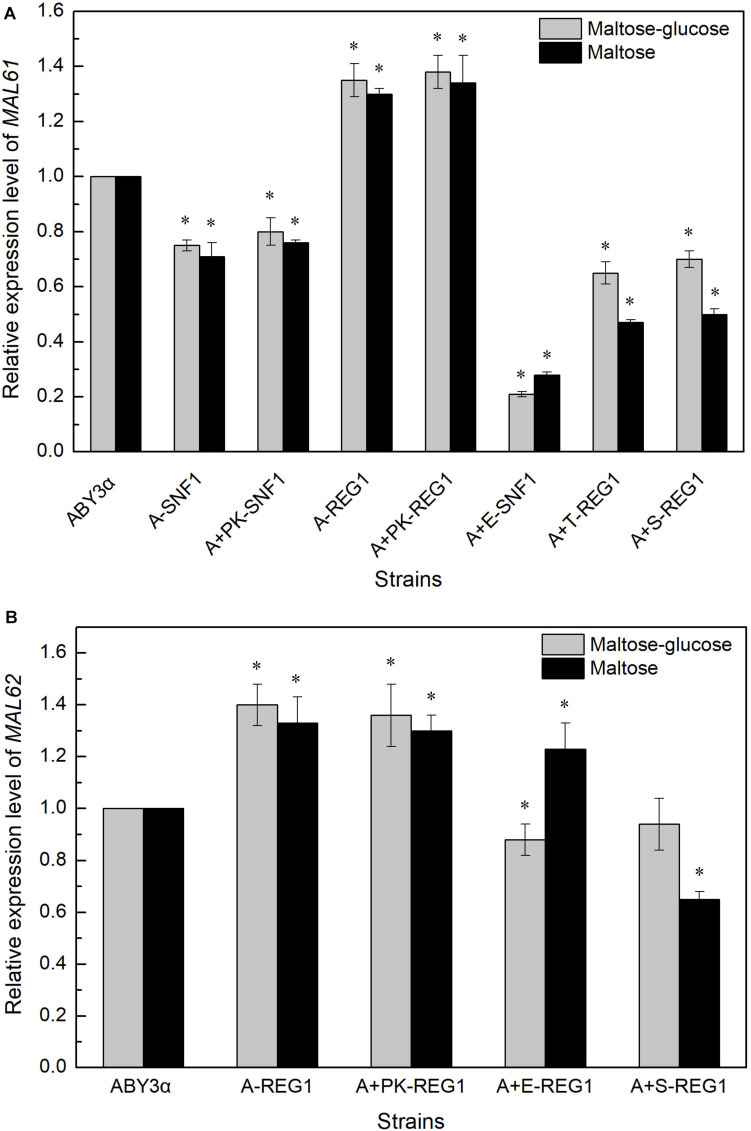
Expression levels of **(A)** *MALx1* and **(B)** *MALx2* in the gene-combination mutants. 2 g fresh yeast was cultured in the LSMLD media and cultures were sampled at 30 min. The expression levels of genes **(A)** *MAL61* and **(B)** *MAL62* were tested using qRT-PCR. ABY3α, the parental strain; A-SNF1, the *SNF1*-deleted mutant; A+PK-SNF1, the *SNF1*-deleted mutant carrying the vector Yep-PK; A-REG1, the *REG1*-deleted mutant; A+PK-REG1, the *REG1*-deleted mutant carrying the vector Yep-PK; A+E-SNF1, the *SNF1*-deleted mutant carrying *ELM1* overexpression; A+E-REG1, the *REG1*-deleted mutant carrying *ELM1* overexpression; A+T-REG1, the *REG1*-deleted mutant carrying *TOS3* overexpression; A+S-REG1, the *REG1*-deleted mutant carrying *SAK1* overexpression. Significant differences of the transformants to the parental strain were confirmed at ^∗^*P* < 0.05.

The *MAL61* mRNA level was tested in the overexpression of *ELM1* combined with the deleted *SNF1* to investigate whether Elm1 regulated the expression of *MALx1* via the Snf1 pathway. Compared with the parental strain, the expression of *MAL61* considerably decreased in strain A+E-SNF1 (Figure 6A), suggesting the possibility of the Elm1-Snf1-Malx1 pathway in the maltose metabolism of baker’s yeast. The downregulated transcription of *MAL* in the overexpression strains suggested the Snf1-independent regulatory pathways by the kinases. Liu et al. (2011) showed that Sak1 interacted with the other protein (mainly referred to Reg1) without relying on Snf1 in glucose. Reg1 is one of the regulatory subunits of the type 1 protein phosphatase of baker’s yeast and regulates glucose repression by targeting the catalytic subunit Glc7 to the corresponding substrates (Tabba et al., 2010). The deletion of *REG1* can increase the expression of *MAL61* and *MAL62*, and enhance the activities of maltose permease and maltase of industrial baker’s yeast (Lin et al., 2015, 2018). Therefore, the expression levels of *MAL61* and *MAL62* were tested in the *REG1-*deleted genetic background. The expression levels of *MAL61* and *MAL62* in strain A+S-REG1 were lower than those of the parental strain in maltose-glucose and maltose (Figure 6). These results suggested the possibility of a Reg1-independent form in the regulation of genes involved in maltose metabolism by Sak1. Similarly, Tos3 regulated the expression of *MAL61* via pathways unrelated to Reg1 under the maltose-glucose and maltose conditions as well as the regulation of *MAL62* by Elm1 (Figure 6). These results indicated that the three kinases differentially regulated the transcription of *MAL* genes via unknown, complex pathways but in a carbon source- and *MAL*-dependent manner.

## Discussion

*Saccharomyces cerevisiae* Elm1, Tos3, and Sak1 kinases are known as the upstream regulators of the Snf1-Mig1 pathway in glucose repression (Hedbacker and Carlson, 2008). In this study, the focus was on the role of the three upstream kinases in the maltose metabolism of baker’s yeast. The results suggested that Elm1, Tos3, and Sak1 played different roles in the regulation of the maltose metabolism of baker’s yeast. Elm1 was necessary for the maltose metabolism of baker’s yeast in maltose and maltose-glucose, and the overexpression of *ELM1* could promote the utilization of maltose. Native Tos3 and Sak1 were essential for yeast cells to adapt glucose inhibition, but high levels of *TOS3* and *SAK1* negatively affected the maltose metabolism.

In this study, the overexpression of *ELM1* alleviated glucose repression and upregulated the expression of *MAL61* and *MAL31* in glucose repression and maltose induction. The increase in maltose uptake by *MAL61* overexpression could facilitate the maltose metabolism and fermentation ability of baker’s yeast in lean dough (Zhang et al., 2015b). The existence of the Elm1-Snf1-Mal61 pathway demonstrated that Elm1 might be one of the dominant upstream regulators in the glucose repression of maltose. The inferior transcription of *MAL62* and *MAL32* could affect maltose hydrolysis and delay the release of CO_2_ in the initial lean dough fermentation in *ELM1* overexpression, and then, high CO_2_ was produced with an upregulated *MAL32* in a glucose derepression condition. These findings demonstrated the differential regulation on *MAL* genes by Elm1. Snf1 is a positive regulator of maltose metabolism in baker’s yeast. Elm1 functions in many cellular activities of yeast in addition to glucose repression, and multiple pathways intersect in response to signals (Souid et al., 2006; Ye et al., 2008; Casamayor et al., 2012). Therefore, Elm1 might regulate the expression of *MALx2*, even *MALx1*, via other unknown pathways rather than relying on Snf1.

The overexpression of *TOS3* and *SAK1* repressed the uptake of maltose with a downregulated transcription of *MAL61/MAL31* in maltose-glucose, thereby inhibiting maltose metabolism and lean dough fermentation. However, native Tos3 and Sak1 were necessary to adapt the glucose repression because the deletion of *TOS3* and *SAK1* decreased maltose metabolism in maltose-glucose and the initial lean dough fermentation. The increased expression of *MAL32* without glucose repression could contribute to the release of CO_2_ in the late lean dough fermentation in *TOS3* and *SAK1* deletions. These findings did not contradict the view that Sak1 is the central upstream kinase involved in the regulation of Snf1 in glucose repression (Hedbacker et al., 2004) and suggested Snf1-independent pathways other than the Reg1-dependent form by the kinases. The regulation of the kinases on maltose metabolism differed from that of invertase in glucose limitation (McCartney et al., 2005). Therefore, the three protein kinases regulated carbon sources metabolism in a signal-dependent manner, and different responses were produced in the same signal (Hong et al., 2003). The specific regulation pathway of Elm1, Tos3, and Sak1 in the maltose metabolism of baker’s yeast needs to be studied further.

The function of the three protein kinases in the regulation of cell growth differed from that in the regulation of maltose metabolism. The overexpression of *ELM1* improved the growth of baker’s yeast in glucose, confirming the role of Elm1 in the coordination of cell growth in budding yeast (Bouquin et al., 2000; Sreenivasan et al., 2003). A similar effect was caused by *TOS3* overexpression in the same condition. This result showed a discrepancy to the findings of Kim et al. (2005), who reported that the mutation of *TOS3* negatively affects the growth of a laboratory *S. cerevisiae* strain in a non-fermentable carbon source with no effect on glucose and raffinose. This finding may be attributed to the discrepancy of yeast strains and the test method used. The overexpression of *SAK1* enhanced the growth of baker’s yeast in glucose and maltose. This finding was consistent with the results of Raab et al. (2011), who analyzed in glucose and non-fermentable carbon source conditions, and suggested the dominant role of Sak1 in the regulation of cell growth in maltose. The increase in growth cannot compensate for the reduction in maltose metabolism caused by downregulated *MAL* in *TOS3* and *SAK1* mutants.

**FIGURE 7:**
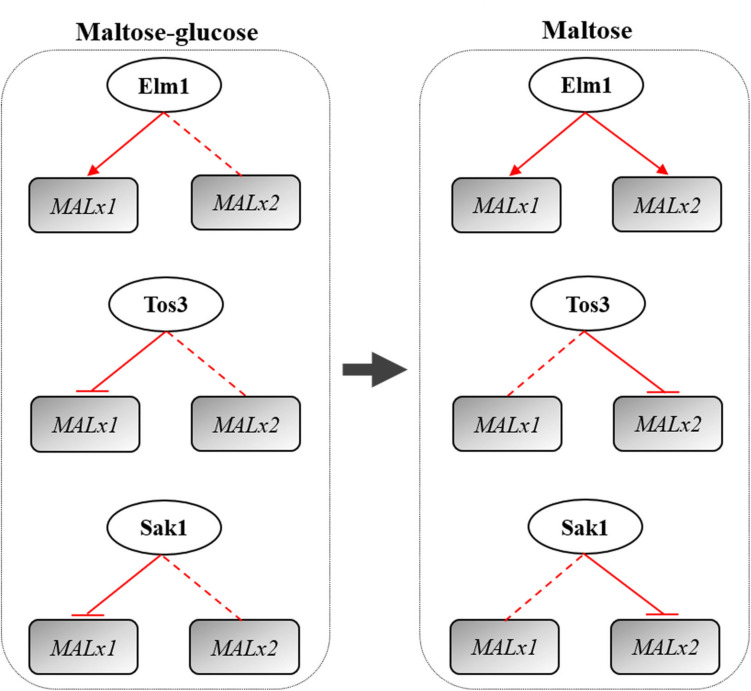
Regulation of the kinases Elm1, Tos3, and Sak1 on *MALx1* and *MALx2* in maltose-glucose and maltose. *x* was the locus and *MAL3* and *MAL6* were analyzed in this study. Red arrow line: positive regulation. Red straight line: native level of the kinase needed. Red flat end line: negative regulation. *MALx2* in the regulation of Tos3 in maltose mainly was *MAL32*. *MALx1* in the regulation of Sak1 in maltose-glucose mainly was *MAL31*. *MALx2* in the regulation of Sak1 in maltose mainly was *MAL32*.

Overall, Elm1, Tos3, and Sak1 played different roles in the regulation of maltose metabolism of baker’s yeast with differential regulations on *MAL* genes (Figure 7). Elm1 was necessary for the maltose metabolism of baker’s yeast in maltose and maltose-glucose, and the overexpression of *ELM1* could enhance the maltose metabolism and lean dough fermentation ability of baker’s yeast by upregulating the transcription of *MAL61* and *MAL31* in maltose and maltose-glucose and the transcription of *MAL62* and *MAL32* in maltose. The native level of *TOS3* and *SAK1* was essential for yeast cells to adapt glucose repression, but the overexpression of *TOS3* and *SAK1* alone negatively affected maltose metabolism largely by repressing the expression of *MAL61*/*MAL31* in maltose-glucose and the expression of *MAL32* in maltose. Moreover, the three upstream kinases might regulate maltose metabolism via the Snf1-parallel pathways with a carbon source-dependent manner. These findings provided a new perspective for breeding industrial yeasts with rapid maltose metabolism and insights into the study of glucose repression in other carbon sources.

## Data Availability Statement

The original contributions presented in the study are included in the article/Supplementary Material, further inquiries can be directed to the corresponding author.

## Author Contributions

XL conceived and designed the research, and drafted the manuscript. XY and LM preformed the experiments. H-YJ participated in the data analysis. X-PH and C-FL revised the manuscript. All authors read and approved the manuscript.

## Conflict of Interest

The authors declare that the research was conducted in the absence of any commercial or financial relationships that could be construed as a potential conflict of interest.
