# Supplementary material for: Role of Elm1, Tos3, and Sak1 Protein Kinases in the Maltose Metabolism of Baker’s Yeast
Source: Front Microbiol. 2021 Jun 1;12:665261. doi: 10.3389/fmicb.2021.665261 (PMC8204090; doi:10.3389/fmicb.2021.665261)
Supplement: Supplementary file 1 [file Data_Sheet_1.docx]

**Supplementary** **Table 1. Specific growth rate (h^-1^) and biomass yield (g/L) of the gene-deleted mutants.**

| Strains | Specific growth rate | Biomass yield | Specific growth rate | Biomass yield |
| --- | --- | --- | --- | --- |
|  | Glucose | | Maltose | |
| ABY3α | 0.512 ± 0.002 | 8.24 ± 0.12 | 0.460 ± 0.002 | 8.08 ± 0.09 |
| A-E | 0.404 ± 0.003^*^ | 7.92 ± 0.13 | 0.323 ± 0.002^*^ | 7.74 ± 0.10^*^ |
| A-T | 0.418 ± 0.003^*^ | 8.24 ± 0.12 | 0.374 ± 0.002^*^ | 7.93 ± 0.07 |
| A-S | 0.405 ± 0.005^*^ | 7.92 ± 0.10 | 0.396 ± 0.003^*^ | 7.86 ± 0.08^*^ |

ABY3α: the parental strain; A-E: the *ELM1*-deleted mutant; A-T: the *TOS3*-deleted mutant; A-S: the *SAK1*-deleted mutant.

Significant differences of the mutants to the parental strain were confirmed at **p* < 0.05.


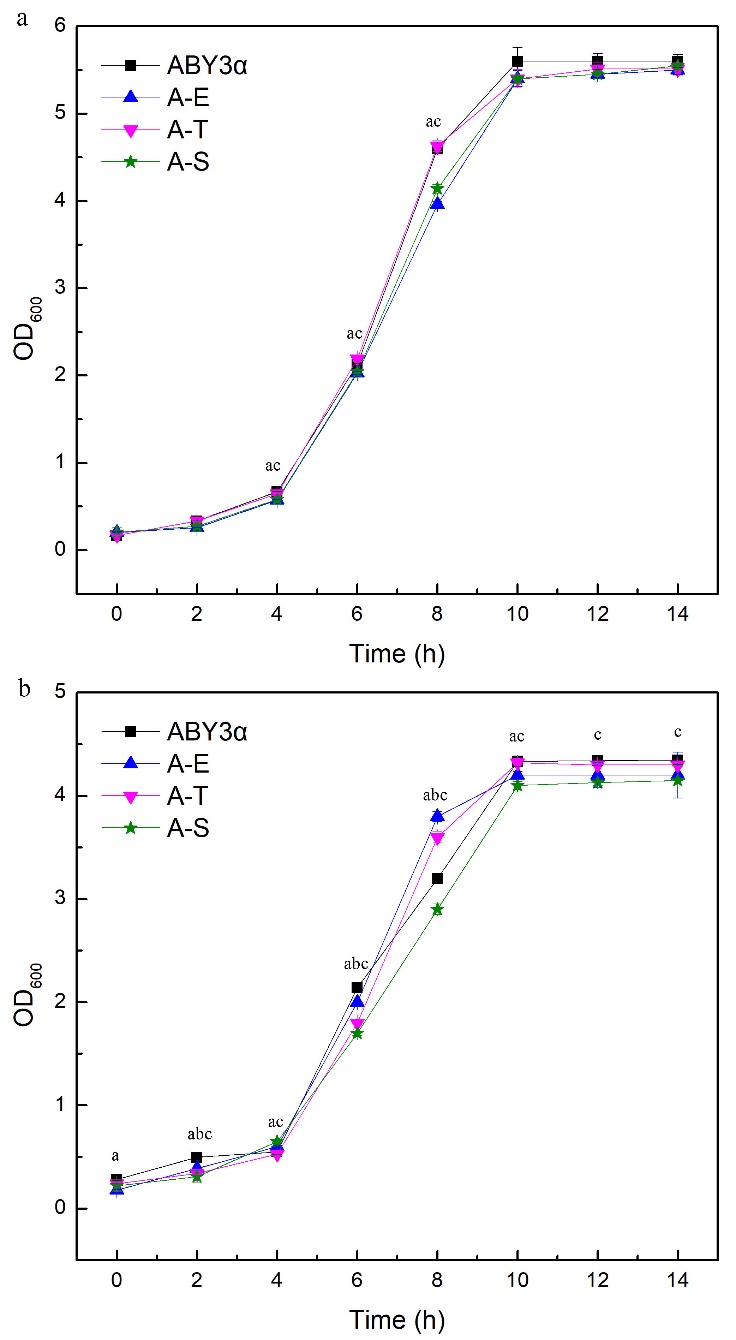


**Supplementary Figure 1. Growth curves of the gene-deleted mutants.** Growth curves were monitored in **a** 2% glucose (YEPD medium) and **b** 2% maltose (YEPM medium consisted of 20 g/L maltose, 20 g/L peptone, and 10 g/L yeast extract) conditions at appropriate time intervals at 30℃. ABY3α: the parental strain; A-E: the *ELM1*-deleted mutant; A-T: the *TOS3*-deleted mutant; A-S: the *SAK1*-deleted mutant. Significant differences of the mutants A-E, A-T, and A-S to the parental strain were confirmed at ^a^*p* < 0.05, ^b^*p* < 0.05, and ^c^*p* < 0.05, respectively.


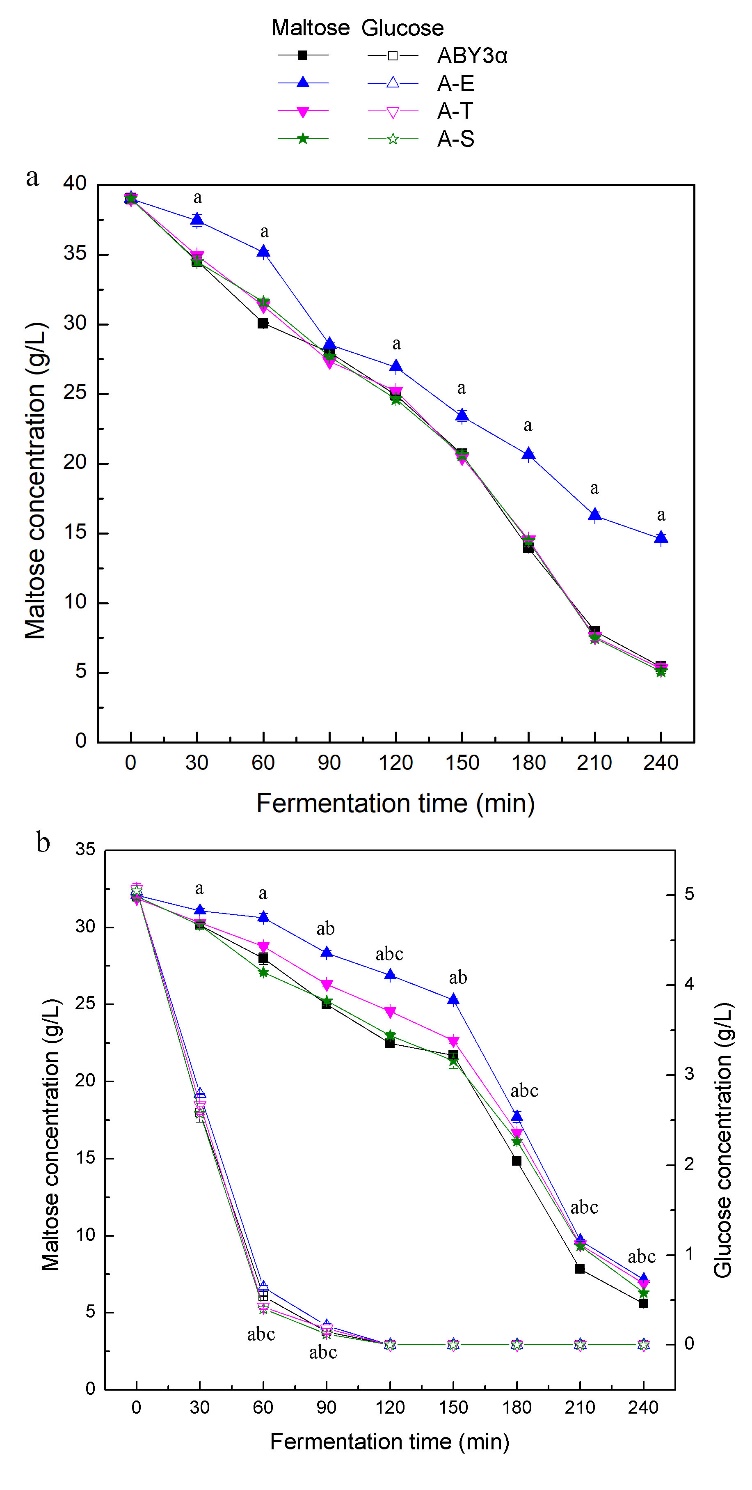


**Supplementary Figure 2. Sugar consumption of the gene-deleted mutants.** 2 g fresh yeast was cultured in the **a** maltose and **b** maltose-glucose LSMLD media at 30°C and 1 mL cultures were sampled at a certain interval. ABY3α: the parental strain; A-E: the *ELM1*-deleted mutant; A-T: the *TOS3*-deleted mutant; A-S: the *SAK1*-deleted mutant. Significant differences of the mutants A-E, A-T, and A-S to the parental strain were confirmed at ^a^*p* < 0.05, ^b^*p* < 0.05, and ^c^*p* < 0.05, respectively.


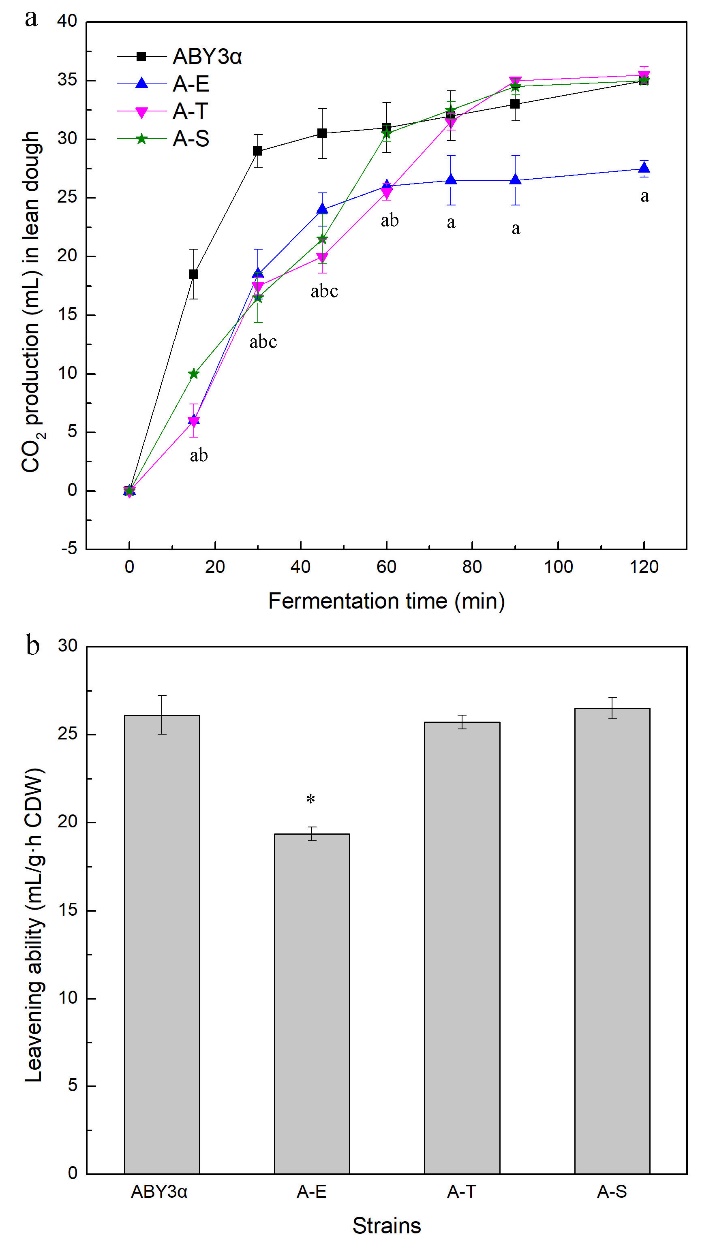


**Supplementary Figure 3. CO_2_ production of the gene-deleted mutants in lean dough.** **a** Mixed dough was placed into a graduated cylinder, and CO_2_ amounts were recorded at 30°C for 120 minutes. Significant differences of the mutants A-E, A-T, and A-S to the parental strain were confirmed at ^a^*p* < 0.05, ^b^*p* < 0.05, and ^c^*p* < 0.05, respectively. **b** Leavening ability was determined by CO_2_ production per hour per gram (dry weight) of yeast cells. ABY3α: the parental strain; A-E: the *ELM1*-deleted mutant; A-T: the *TOS3*-deleted mutant; A-S: the *SAK1*-deleted mutant. Significant differences of the mutants to the parental strain were confirmed at ^*^ *P* < 0.05.


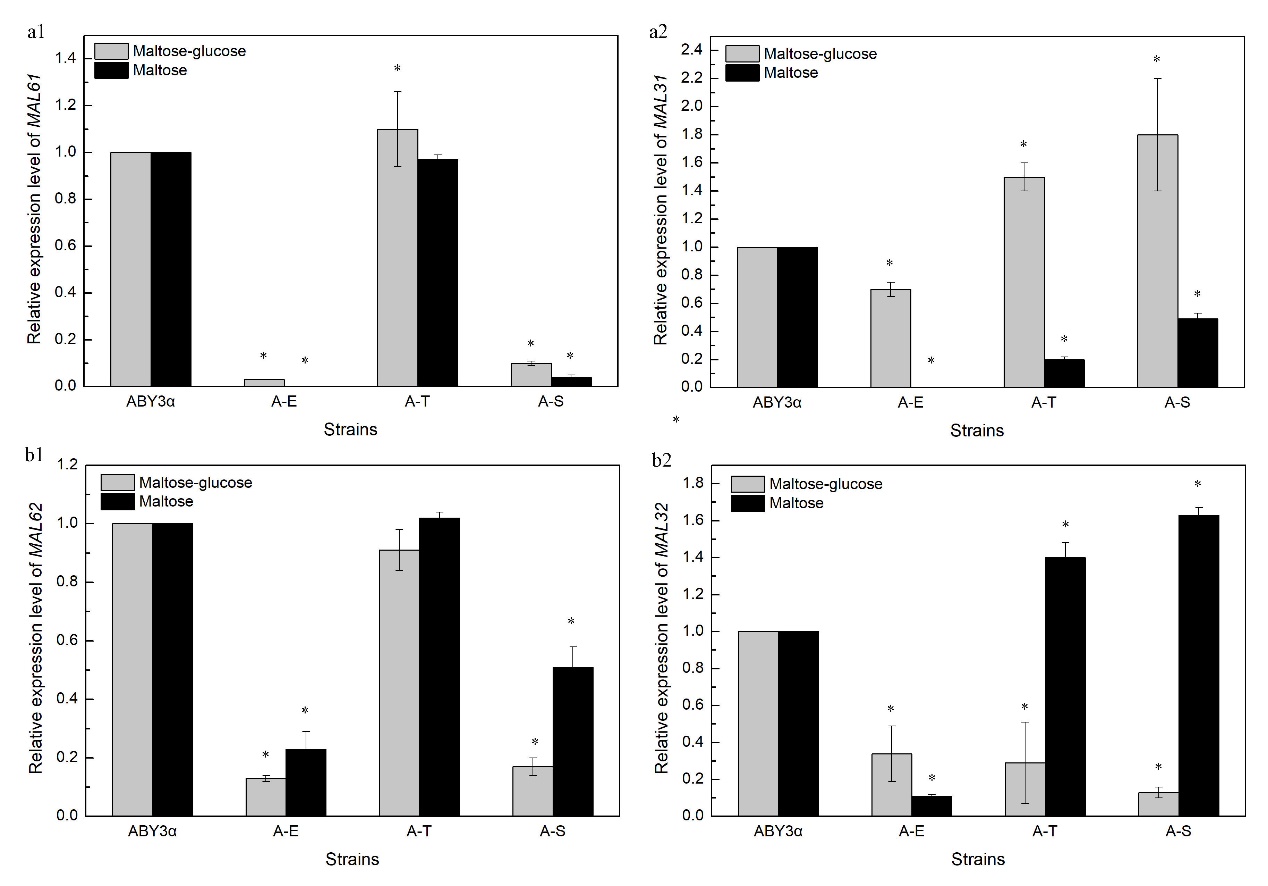


**Supplementary Figure 4. Expression levels of a *MALx1* and b *MALx2* in the gene-deleted mutants.** 2 g fresh yeast was cultured in the LSMLD media and cultures were sampled at 30 min. The expression levels of genes **a1** *MAL61*, **a2** *MAL31* and **b1** *MAL62*, **b2** *MAL32* were tested using qRT-PCR. ABY3α: the parental strain; A-E: the *ELM1*-deleted mutant; A-T: the *TOS3*-deleted mutant; A-S: the *SAK1*-deleted mutant. Significant differences of the mutants A-E, A-T, and A-S to the parental strain were confirmed at ^*^ *P* < 0.05.
